# The Characterization of Subcutaneous Adipose Tissue in Sunit Sheep at Different Growth Stages: A Comprehensive Analysis of the Morphology, Fatty Acid Profile, and Metabolite Profile

**DOI:** 10.3390/foods13040544

**Published:** 2024-02-09

**Authors:** Yunfei Han, Xige He, Yueying Yun, Lu Chen, Yajuan Huang, Qiong Wu, Xia Qin, Haiyan Wu, Jindi Wu, Rina Sha, Gerelt Borjigin

**Affiliations:** 1 College of Food Science and Engineering, Inner Mongolia Agricultural University, Huhhot 010018, China; hanyunfei122@163.com (Y.H.); hexige1212@163.com (X.H.); chenluuu0618@163.com (L.C.); huangyajuan2008@163.com (Y.H.); qinxia98@163.com (X.Q.); 15771395332@139.com (H.W.); wujindi2020@imau.edu.cn (J.W.); spsharina@imau.edu.cn (R.S.); 2 School of Life Science and Technology, Inner Mongolia University of Science and Technology, Baotou 014010, China; yunyueying1999@163.com; 3 Ke Er Qin You Yi Front Banner Administration for Market Regulation, Xing’an League 137400, China; moqiqing163@163.com

**Keywords:** Sunit sheep, subcutaneous fat, lipid, metabolomic, adipocyte

## Abstract

Adipose tissue is a crucial economically significant trait that significantly influences the meat quality and growth performance of domestic animals. To reveal the changes in adipose tissue metabolism during the growth of naturally grazing sheep, we evaluated the thickness, adipocyte morphology, fatty acid profile, and metabolite profile of subcutaneous adipose tissue (SAT) from naturally grazing Sunit sheep at 6, 18, and 30 months of age (referred to as Mth-6, Mth-18, and Mth-30, respectively). The fat thickness and adipocyte number were significantly increased with the growth of the sheep (*p* < 0.05), and the increase of which from Mth-18 to Mth-30 was less than that from Mth-6 to Mth-18. Additionally, the alpha-linolenic acid metabolism was enhanced and fatty acid (FA) elongation increased with growth. The metabolomic analysis revealed 76 differentially expressed metabolites (DEMs) in the SAT in different growth stages. Interestingly, we observed elongation of FAs in lipids correlated with sheep growth. Furthermore, the expression of acylcarnitines was downregulated, and fatty acid amides, aspartic acid, acetic acid and phosphocholine were upregulated in Mth-18 and Mth-30 compared to Mth-6. Altogether, the study found that the difference in SAT in Mth-6 was great compared to Mth-18 and Mth-30. An increase in fat deposition via adipocyte proliferation with the growth of the sheep in naturally grazing. The DEMs of acylcarnitines, fatty acid amides, aspartic acid, acetic acid, and phosphocholine emerged as potential key regulators of adipose tissue metabolism. These findings illustrate the variation in and metabolic mechanism of sheep adipose tissue development under natural grazing, thus providing valuable insights into improving the edible quality of sheep meat and developing the mutton sheep industry.

## 1. Introduction

Adipose tissue, an essential and dynamic endocrine organ, plays a crucial role in animal survival under extreme conditions, such as cold, heat, food scarcity, and high altitude [1,2]. It is distributed throughout various regions of the sheep’s body, including the subcutaneous, visceral, intramuscular, intermuscular, and tail areas. Subcutaneous adipose tissue (SAT) exhibits the widest distribution among these tissues, and is closely associated with carcass characteristics, such as intramuscular fat content, tenderness, and flavor [3,4,5]. Moreover, SAT provides protection against cold shortening and drip loss during the cooling (0–4 °C) of the carcass [6]. Similar to the functions of intermuscular and intramuscular fat in meat quality attributes, SAT affects the taste, flavor, and nutritional value of the edible meat.

Adipose tissues exhibit remarkable changes in their size in response to energy surplus or deficit, displaying a high degree of plasticity and dynamism [7]. This phenomenon is referred to as adipose tissue remodeling, encompassing adipocyte hyperplasia and hypertrophy, characterized by an increase in both the number of adipocytes and accumulation of lipid content within these cells [8,9]. In recent years, metabolomics has emerged as a valuable tool for investigating fat metabolism. Du et al. [10] investigated the lipidomic profiles of SAT, visceral adipose tissue (VAT), and abdominal adipose tissue (AAT) in Huaxi cattle, suggesting that VAT and AAT exhibited similar lipid compositions. However, SAT had higher levels of monounsaturated fatty acids (MUFAs) and triglycerides (TGs) compared to VAT and AAT. Xiong et al. [11] proved the importance of glycerolipid metabolism and the functions of insulin and cholesterol in the regulation of yak fat deposition. Additionally, another study by Xiong et al. [12] revealed that fat metabolism in yaks is influenced by carbohydrate, fatty acid, and amino acid metabolism through the tricarboxylic acid (TCA) cycle. Lan et al.’s study [13] involving pigs showed that metabolites such as epinephrine, cAMP, arachidonic acid, oleic acid, linoleic acid, and docosahexaenoic acid exhibited age-specific effects and played important roles in lipolysis, fat accumulation, and fatty acid composition. These studies provided insights into the distinct characteristics of different adipose tissues and identified several metabolites and metabolic pathways associated with fat deposition. However, the metabolism of adipose tissue differs depending on the species [14], age [15], anatomical location [10], and feeding conditions [11]. Our previous studies have revealed that growth affects tail fat metabolism in sheep. Tail fat metabolism is more active during the early stages of sheep growth, and this gradually decreases as the animal ages [2,15]. However, alterations in SAT metabolism during the different growth stages remain poorly understood, especially for naturally grazing sheep.

The Sunit sheep is a breed of Mongolian sheep; this species exhibits traits such as cold tolerance, drought resistance, rapid growth and development, high vitality, tender flesh, and excellent flavor that contribute to its popularity among consumers [2]. Typically, companies prefer slaughtering sheep under the age of one year (especially lambs around 6 months old) for optimal commercial value [16]. However, local inhabitants tend to slaughter sheep over one year old and even three years old for consumption and roll meat with fatty tissue, which is closely associated with SAT deposition and the edible quality of the meat. We conducted a comprehensive comparative analysis of the SAT in Sunit sheep under natural grazing conditions, focusing on fat thickness, adipocyte morphology, fatty acid profile, and metabolite profile across three different growth stages: 6 months (Mth-6), 18 months (Mth-18), and 30 months (Mth-30). The integration of phenotype and metabolomic analyses enables a more comprehensive investigation into the variations and underlying metabolic mechanisms of SAT in Sunit sheep during different growth stages under natural grazing conditions. This study can provide new insights into the ovine adipose tissue metabolic mechanism during development under natural grazing, which has significant implications for enhancing the sheep meat quality traits and advancing the mutton sheep industry.

## 2. Materials and Methods

### 2.1. Sample Collection

The raw materials for this omics experiment were obtained from castrated (within 30 days of birth) rams during three different growth stages: 6 (Mth-6, *n* = 6, average weight: 29.43 kg ± 0.90), 18 (Mth-18, *n* = 6, average weight: 48.57 kg ± 1.32), and 30 (Mth-30, *n* = 6, average weight: 56.97 kg ± 1.71) months of age. All Sunit sheep were selected from the same herd, and raised under natural grazing conditions in the Xilingol grasslands of the Sunit banner, Inner Mongolia, where the forage species are abundant [17]. Following slaughter at the local abattoir, the subcutaneous adipose tissue (SAT, backfat at the 12th rib) was sampled, frozen in liquid nitrogen, and stored at −80 °C.

### 2.2. Fat Thickness Measurement

Naturally grazing castrated rams of the ages Mth-6, Mth-18, and Mth-30 (50 sheep per growth stage) were selected after slaughter at the local abattoir. The height of the backfat cross-section at the 12th rib was measured in mm with vernier calipers [18].

### 2.3. Histological Analysis

In accordance with the method outlined by Zheng et al. [19], adipose tissue was cut into pieces that were 1 cm × 1 cm × 0.5 cm in size, and the pieces were fixed overnight with 4% paraformaldehyde in phosphate-buffered saline. The tissue samples were then transferred to 70% ethanol and embedded in paraffin. Samples were cut into 5 μm sections, stained with hematoxylin–eosin (H&E), and examined via a light microscopy. Adipocyte diameter and area were determined using Image-Pro Plus 6.0 software (Media Cybernetics, Rockville, ML, USA).

### 2.4. Extraction and Analysis of Fatty Acids

FAs were analyzed using gas chromatography. Total fats were extracted from the samples (0.2 g from each sample) with chloroform/methanol (2:1, *v*/*v*) and quantified gravimetrically according to the method outlined by Folch [20]; then, fatty acid methyl esters (FAMEs) were obtained from the total fats using the acid-catalyzed transesterification procedure described by Christie [21]; FAMEs were then quantified using gas chromatography supplied with a hydrogen flame ionization detector and a capillary column (100 m × 0.25 mm × 0.20 µm). The FAMEs were identified by matching the corresponding retention times (RTs) with that of FAME mix standards (Supelco 37 Component FAME Mix, Sigma-Aldrich, Darmstadt, Germany). FAMEs were quantified using known internal standard (undecanoic acid methylester, Sigma-Aldrich, Germany) concentrations.

### 2.5. Extraction and Analysis of Metabolites

Metabolite extraction and mass spectrometer-based metabolomic analysis were performed following the methods described by Li et al. [22]. The metabolites were extracted from the samples (0.1 g from each sample) with 50% methanol buffer. The extraction mixture was stored overnight at −20 °C. After centrifugation, the supernatants were stored at −80 °C prior to the liquid chromatography–mass spectrometry analysis.

All samples were acquired using the LC–MS system following machine orders. Firstly, all chromatographic separations were performed using an ultra-performance liquid chromatography (UPLC) system (SCIEX, Warrington, UK). An ACQUITY UPLC T3 column (100 mm × 2.1 mm, 1.8 µm, Waters, Wilmslow, UK) was employed for the reversed-phase separation. A high-resolution tandem mass spectrometer, TripleTOF5600plus (SCIEX, Warrington, Cheshire, UK), was used to detect metabolites eluted from the column. The Q-TOF was operated under both positive and negative ion modes.

The data were collected using Proteowizard software; converted into mzXML format; and then the peak alignment, retention time correction, and peak area were obtained using XCMS software. The data extracted using XCMS were identified using CAMERA and metaX. An in-house MS2 database was used to identify metabolites, which were finally analyzed.

### 2.6. Statistical Analysis

The experimental data were presented as the mean ± standard deviation. The experimental data were subjected to analysis of variance (ANOVA) using SPSS 16.0 (SPSS, Chicago, IL, USA). Significant difference and highly significant difference were set as *p* < 0.05 and *p* < 0.01, respectively. Principal component analysis (PCA) and cluster analysis were performed using the OmicStudio tools at https://www.omicstudio.cn/tool (accessed on 27 May 2021). Significantly different metabolites were defined with the criteria of fold change >2 or <0.5, *p*-value (*p*) < 0.05, and variable influence in the projection (VIP) > 1. Enrichment pathway analysis was performed for the DEMs using MetaboAnalyst 5.0, with a *p* < 0.05 considered significantly enriched.

## 3. Results

### 3.1. Fat Thickness and Adipocyte Morphology Analysis

**Figure 1:**
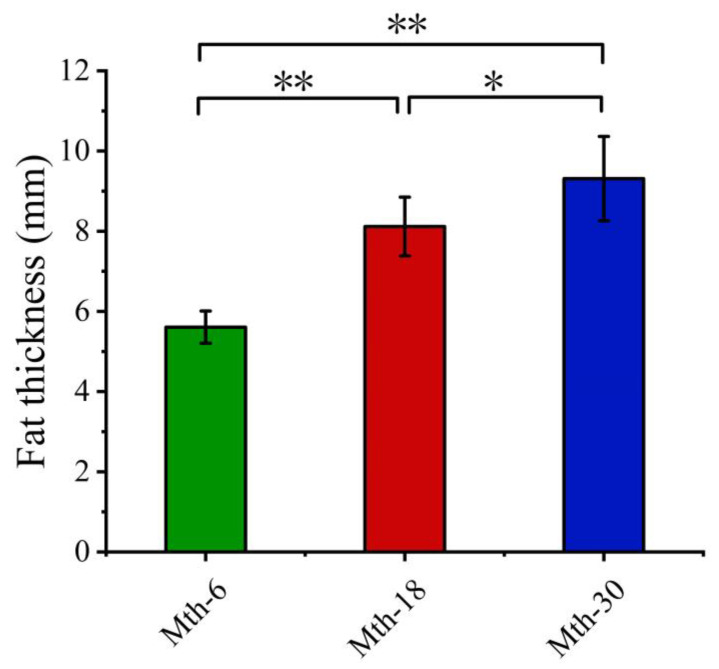
The thickness of subcutaneous adipose tissue in the different growth stages. * *p* < 0.05; ** *p* < 0.01.

**Figure 2:**
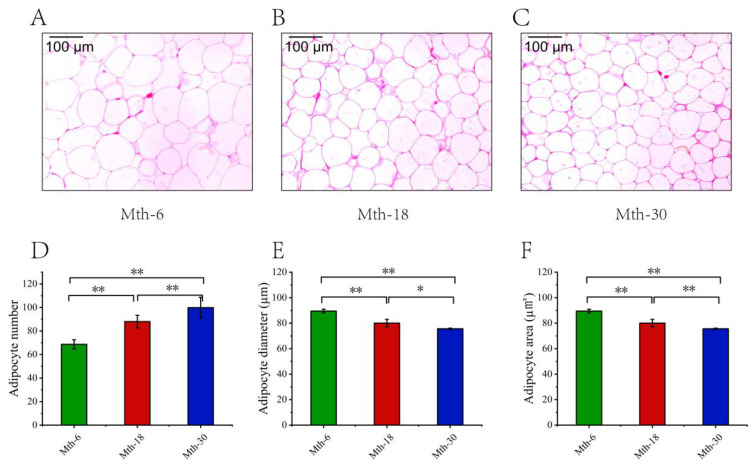
The adipocyte morphology analysis of subcutaneous adipose tissue in the different growth stages. (**A**–**C**) Adipocyte morphology (**A**): Mth-6, (**B**): Mth-18, and (**C**): Mth-30). (**D**) Adipocyte number. (**E**) Adipocyte diameter. (**F**) Adipocyte area. * *p* < 0.05; ** *p* < 0.01.

In this study, the fat thickness increased highly significantly by 2.84 mm comparing Mth-6 to Mth-18 (*p* < 0.01). Furthermore, it showed a significant increase of 1.02 mm when comparing Mth-18 to Mth-30 (*p* < 0.05) (Figure 1). The alterations in the morphology of the adipocytes in the SAT are presented in Figure 2. In the same size field of view, the average adipocyte numbers were 69, 88, and 100 in Mth-6, Mth-18, and Mth-30, respectively; moreover, the adipocyte number was highly significantly increased (*p* < 0.01) with the growth of the sheep. Meanwhile, the individual adipocyte area was highly significantly decreased (*p* < 0.01) with the growth of the sheep (Figure 2D,F). It decreased by 1084.8 μm^2^ in Mth-18 compared to in Mth-6 and 854.0 μm^2^ in Mth-30 compared to in Mth-18. Correspondingly, the individual adipocyte diameter was highly significantly decreased from Mth-6 to Mth-18 (*p* < 0.01) and significantly decreased from Mth-18 to Mth-30 (*p* < 0.05) (Figure 2E). The changes in fat thickness and adipocyte number were relatively slower from Mth-18 to Mth-30 than from Mth-6 to Mth-18.

### 3.2. Fatty Acid Profile Analysis

**Figure 3:**
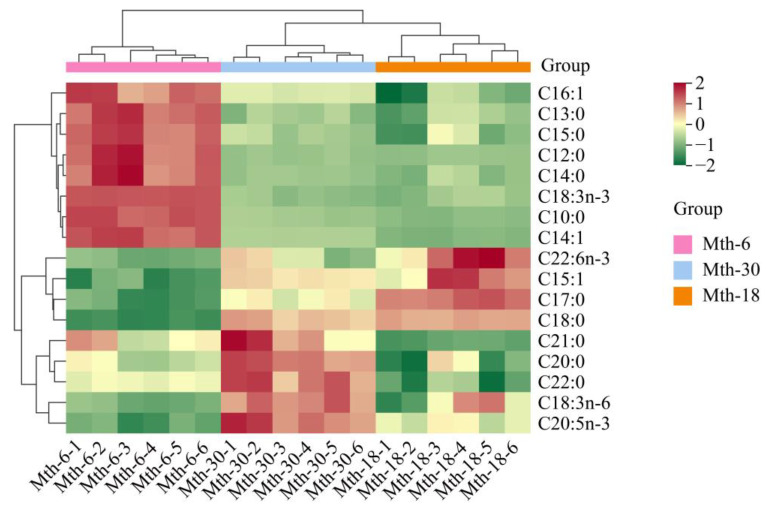
The hierarchical clustering analysis of differentially expressed fatty acids (*p* < 0.05) of subcutaneous adipose tissue in different growth stages.

Regarding the results of the composition of fatty acids (Table 1), the FAs were predominantly palmitic acid (C16:0), stearic acid (C18:0), and oleic acid (9c-C18:1) for the Sunit sheep SAT. The content of saturated fatty acids (SFAs) was higher than that of MUFAs and polyunsaturated fatty acids (PUFAs) in all samples. The content of SFAs was significantly lower in Mth-6 compared to that in Mth-18 and Mth-30, while the content of PUFAs was significantly higher (*p* < 0.05). The PUFA/SFA in Mth-6 was significantly higher than that in Mth-18 and Mth-30 (*p* < 0.05). The content of n-3 FAs in Mth-6 was significantly higher than that in Mth-18 and Mth-30 (*p* < 0.05). The n-6/n-3 was significantly lower in Mth-6 than that in Mth-18 and Mth-30 (*p* < 0.05). In Figure 3, we present the hierarchical clustering analysis of seventeen differentially expressed (DE) FAs in the three growth stages (*p* < 0.05), which are divided into three clusters. The first cluster was the content of fatty acids that were the highest during Mth-6. Most of the fatty acids that were observed were medium- and long-chain saturated fatty acids (MLC-SFAs, including eight- and more-than-eight-carbon FAs), including capric acid (C10:0), dodecanoic acid (C12:0), tridecanoic acid (C13:0), myristic acid (C14:0), pentadecanoic acid (C15:0), etc. The second cluster included heptadecanoic acid (C17:0), pentadecenoic acid (C15:1), C18:0, and docosahexaenoic acid (C22:6n-3), which were relatively highly expressed during Mth-18 and Mth-30. The third cluster mainly comprised very-long-chain fatty acids (VLC-FAs, including 20- and more-than-20-carbon FAs), alpha-linolenic acid (C18:3n-6), eicosapentaenoic acid (C20:5n-3), arachidic acid (C20:0), eicosanoate (C21:0), and behenic acid (C22:0), and these FAs had a higher content in Mth-30 than in Mth-6 and Mth-18. It was found that medium- and long-chain fatty acids (MLC-FAs) were gradually elongated, converting into VLC-FAs with the growth of the sheep. It is noteworthy that three n-3 FAs (alpha-linolenic acid (C18:3n-3), C20:5n-3, and C22:6n-3) were distinctly expressed in different growth stages. In comparison with Mth-6, the content of C18:3n-3 significantly decreased (*p* < 0.05), while the contents of C20:5n-3 and C22:6n-3 significantly increased in Mth-18 (*p* < 0.05).

### 3.3. Metabolite Profile Analysis

#### 3.3.1. Summary of Metabolite Profile Analysis

**Figure 4:**
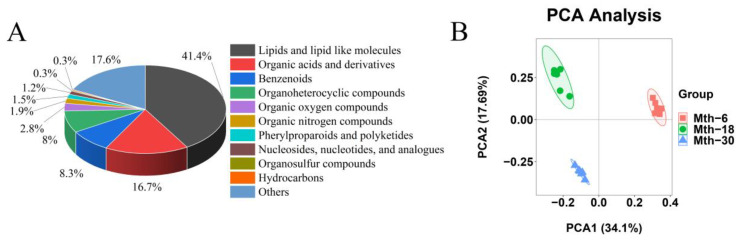
General information for the metabolomic data. (**A**) Classification of metabolites. (**B**) Principal component analysis of the metabolites.

A total of 324 metabolic compounds were identified using an in-house mass spectrometry-based targeted metabolomics approach, which were subsequently included in the analysis and further categorized into 11 distinct species (Figure 4A). The major species identified were lipids and lipid-like molecules (41.4%), organic acids and derivatives (16.7%), benzenoids (8.3%), and organoheterocyclic compounds (8%). The PCA of the metabolite expression profiles is shown in Figure 4B, and the total principal components are represented in Table S1. The six samples for each group were clustered together, and the same group was robustly separated from other groups, suggesting that there were great differences between Mth-6, Mth-18, and Mth-30.

#### 3.3.2. Differential Expression Analysis

**Figure 5:**
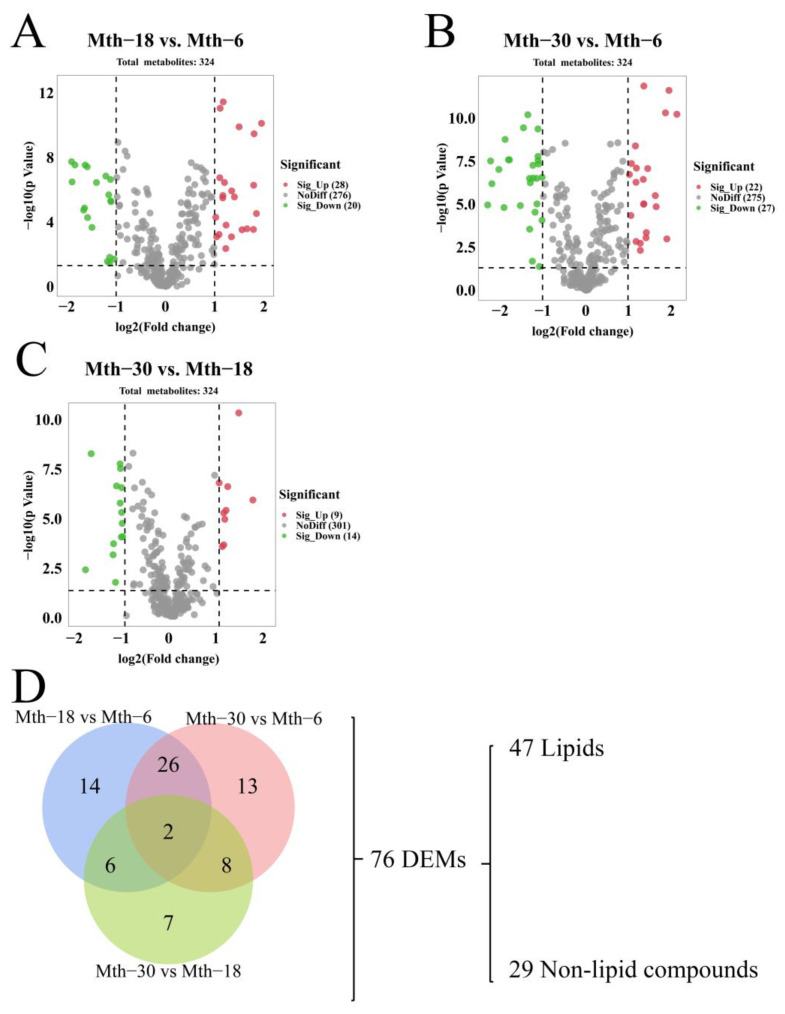
Metabolite differential expression analysis. (**A**–**C**) The volcano plot of the DEMs. (**A**) Mth-18 vs. Mth-6; (**B**) Mth-30 vs. Mth-6; (**C**) Mth-30 vs. Mth-18. (**D**) Venn diagram analysis of the DEMs.

**Figure 6:**
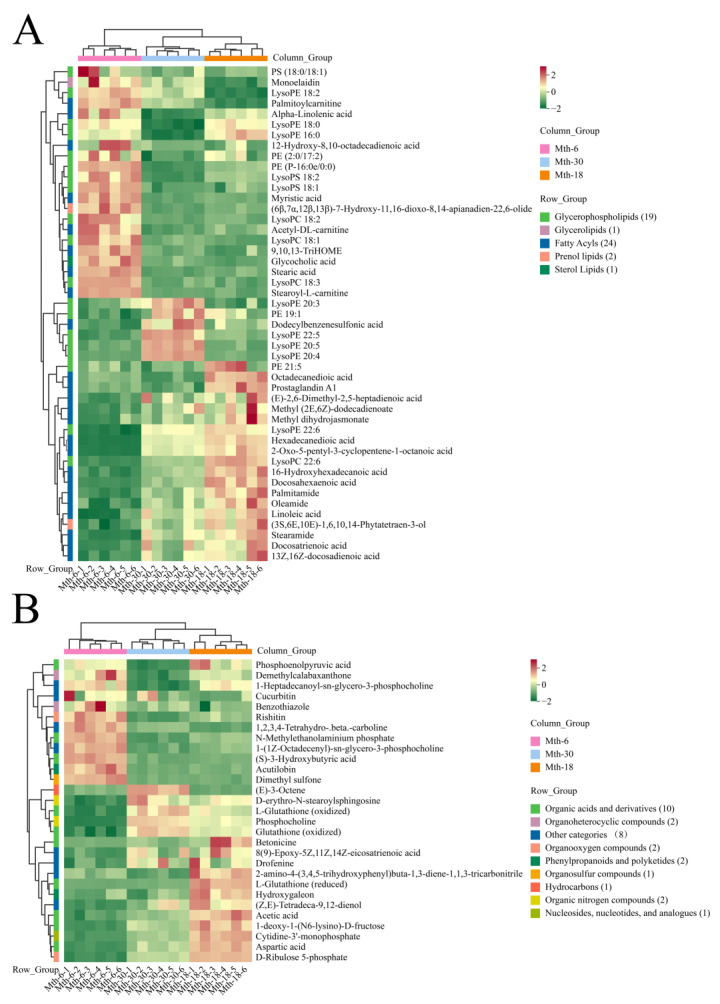
The expression patterns of differentially expressed metabolites. (**A**) The heatmap of the lipids. (**B**) The heatmap of the non-lipid compounds.

We compared metabolites in the SAT of Sunit sheep in three different stages (Mth-18 vs. Mth-6, Mth-30 vs. Mth-6, and Mth-30 vs. Mth-18). As a result, 48 DEMs (20 downregulated and 28 upregulated) were obtained in Mth-18 vs. Mth-6 (Figure 5A). In the Mth-30 vs. Mth-6 comparison, 49 DEMs (27 downregulated and 22 upregulated) were identified (Figure 5B). The lowest number of DEMs was obtained for the Mth-30 vs. Mth-18 comparison, with a total of 23 DEMs (14 downregulated and 9 upregulated) (Figure 5C). Most of the overlapping DEMs were found between Mth-18 vs. Mth-6 and Mth-30 vs. Mth-6 (Figure 5D). A total of 76 DEMs were identified in the three comparison groups, of which the highest numbers were lipid and lipid-like molecules. The 76 DEMs were categorized into two groups for subsequent analysis: 47 DE lipids and 29 DE non-lipid compounds. The expression patterns and cluster analysis of the DE lipids are illustrated in Figure 6A. A total of 22 DE lipids (including stearoylcarnitine, palmitoylcarnitine, acetyl-DL-carnitine, etc.) exhibited a downregulated trend from Mth-6 to Mth-18 and Mth-30. Conversely, 25 DE lipids (such as oleamide, palmitamide, and stearamide) showed the upregulated trend from Mth-6 to Mth-18 and Mth-30. In addition, we compared the co-DE FAs (*p* < 0.05) in FA analysis and metabolomic analysis; the trends of C14:0, C18:3n-3, and C22:6n-3 were consistent in two experimental results, which also demonstrated the reliability of the data. The expression patterns and cluster analysis of the DE non-lipid compounds are illustrated in Figure 6B. Twelve DE non-lipid compounds were clustered with an upregulated trend, and 17 DE non-lipid compounds were clustered with a downregulated trend from Mth-6 to Mth-18 and Mth-30. We found that the three different groups were clearly divided into two further groups, suggesting that there are great differences between lamb (Mth-6) and adult sheep (Mth-18 and Mth-30), which supports the results of the fat thickness, adipocyte morphology, and fatty acid analyses mentioned above.

#### 3.3.3. Enrichment Pathway Analysis of Differentially Expressed Metabolites

**Figure 7:**
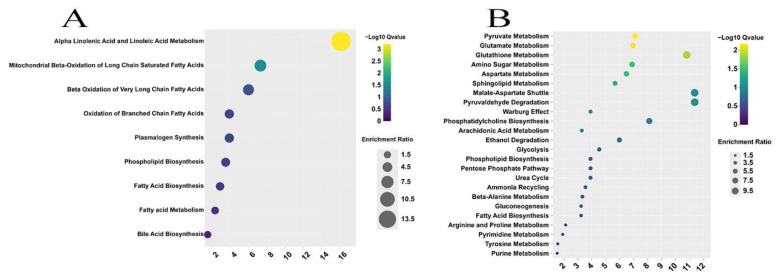
Enrichment analysis of the DEMs. (**A**) Lipid analysis. (**B**) Non-lipid compound analysis.

**Figure 8:**
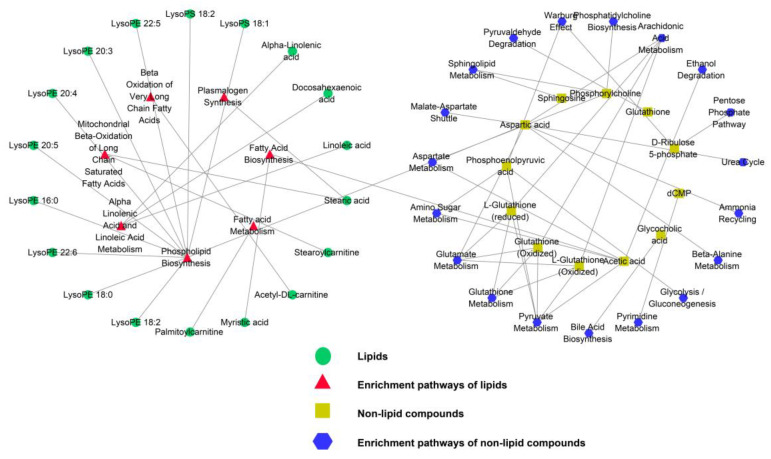
The network of metabolites and enrichment pathways. Green circles represent lipids; red triangles represent the enrichment pathways of lipids; yellow squares represent non-lipid compounds; blue hexagons represent the enrichment pathways of non-lipid compounds.

To study the changing metabolic pathways with the growth of the sheep, enrichment pathway analysis of DE lipids (Figure 7A) and non-lipid compounds (Figure 7B) was performed using MetaboAnalyst 5.0. The DE lipids were enriched in a total of nine pathways, two of which, namely alpha-linolenic acid and linoleic acid metabolism and mitochondrial beta-oxidation of long-chain saturated fatty acids, were significantly enriched (*p* < 0.05). The DE non-lipid compounds were enriched in twenty-four pathways, six of which were significantly enriched (*p* < 0.05), including pyruvate metabolism, glutathione metabolism, glutamate metabolism, amino sugar metabolism, aspartate metabolism, and sphingolipid metabolism. Additionally, metabolic pathways, such as glycolysis/gluconeogenesis, bile acid biosynthesis, and the pentose phosphate pathway, were found to be closely linked to fat metabolism. Interaction networks were constructed to illustrate the molecular relationship between the DEMs and the enrichment pathways associated with them (Figure 8).

## 4. Discussion

The deposition of adipose tissue has a significant impact on the edible quality of sheep meat and the growth performance of sheep. Therefore, it is imperative to investigate the variation and metabolic mechanisms associated with adipose tissue development in order to advance the development of the mutton sheep industry. Under natural grazing conditions, the adipose tissue metabolism of sheep during different growth stages is affected by a number of potential factors, such as grazing periods [23], energy consumption levels [24], cold exposure [25], and physical activity [26]. However, metabolic changes during the SAT development of Sunit sheep under natural grazing have not been reported. In this study, we analyzed the fat thickness, adipocyte morphology, fatty acid profile, and metabolite profile of the SAT of Sunit sheep in three different growth stages (Mth-6, Mth-18, and Mth-30) to explore the variation in and metabolic mechanism of adipose tissue development.

SAT deposition is a necessary stage in the domestic animal fattening process. In this study, subcutaneous fat thickness increased with the growth of the sheep. Fat deposition occurs through adipocyte hypertrophy and/or hyperplasia [27]. From the analysis of adipocyte morphology, the adipocyte number increased, and the individual adipocyte diameter and area decreased with the growth of the sheep. These results revealed the increase in SAT deposition with the growth of natural grazing sheep due to adipocyte hyperplasia rather than hypertrophy. Xu et al.’s study [7] found that the enlarged energy storage capacity of fat-tailed sheep is largely due to an increase in adipocyte number; this result is similar to ours. During early development in goats, fat deposition is mainly caused by adipocyte hyperplasia [28]. In this study, the increase in fat thickness and adipocyte hyperplasia exhibited a relatively smaller magnitude from Mth-18 to Mth-30 compared to that observed from Mth-6 to Mth-18. In other words, the adipocytes in the SAT exhibited a proliferative phase from Mth-6 to Mth-30, and cell proliferation was relatively slow after Mth-18. Fat deposition was related to the variation in metabolism during the growth of the sheep, and its regulation mechanism needs to be investigated further.

FAs, as a crucial constituent of adipose tissue, contribute to flavor, nutritional value, and physiological function [29,30]. According to the results we obtained from the fatty acids analyzed in this study, C16:0, 9c-C18:1, and C18:0 were the most abundant in the SAT. Wang et al.’s study [31] showed that SAT from Sunit sheep was mainly composed of C16:0, C18:0, and C18:1; this result is similar to ours. SFAs were the primary FAs in the SAT. Due to the hydrogenation process of UFAs catalyzed by rumen microflora, ruminant animals exhibit significantly reduced levels of PUFAs [32,33]. The PUFA/SFA, as well as that of n-6/n-3, are two important parameters in the evaluation of meat lipids. The PUFA/SFA was higher, and the n-6/n-3 was lower in Mth-6 than that in Mth-18 and Mth-30. These results implied that the fatty acid composition of Mth-6 was healthier than that in the two groups. The expression pattern analysis of DE FAs implied that the carbon chain in FAs was gradually elongated from Mth-6 to Mth-18 and Mth-30. The elongation of FAs affects the flavor and nutritive value [29], and facilitates the formation of complex lipids [34]. The expression of C17:0 was upregulated from Mth-6 to Mth-18 and Mth-30. Peter et al.’s study [29] showed that C17:0 increases with animal age in sheep as found in the current study. 4-methyloctanoic (MOA) and 4-methylnonanoic (MNA) acids have been implicated as the main compounds responsible for mutton flavor [35]. Watkins and Frank’s study [36] found that C17:0 was associated with MOA and MNA in sheep fat, and that C17:0 was linearly related to the two compounds. The expression of C17:0 is more beneficial for the formation of flavor compounds in mutton (Mth-18 and Mth-30) than that in lamb (Mth-6). It is noteworthy that the expression of three n-3 FAs (C18:3n-3, C20:5n-3, and C22:6n-3) varied across the different growth stages. Specifically, compared to Mth-6, the content of C18:3n-3 decreased, while that of C20:5n-3 and C22:6n-3 increased in Mth-18 and Mth-30. These results suggest that growth may accelerate the metabolism of n-3 FAs. The expression of C18:3n-6 was upregulated from Mth-6 to Mth-18 and Mth-30, which is more beneficial for human health from Mth-18 to Mth-30 than in Mth-6. A total of 76 DEMs were detected in the different growth stages, including 47 lipids and 29 non-lipid compounds. Lipids serve crucial roles as energy carriers, membrane components, and signaling molecules [37]. In the present study, the lipid molecules of the SAT were further divided into five classes, comprising 24 fatty acyls, 19 glycerophospholipids (GPs), 1 glycerolipid, 2 sterol lipids, and 1 sterol lipid. The expression of fatty acyls and GPs most prominently changed during the development of SAT in sheep. For the analysis of fatty acyls, the FA expression levels of C14:00 and C18:3n-3 were highest during Mth-6, and C22:2, C22:6n-3, and C22:3 were upregulated in Mth-18 and Mth-30. The results also revealed that FAs were gradually elongated and desaturated from Mth-6 to Mth-18 and Mth-30. GPs are polar lipids that are ubiquitous in all tissues as they are essential components of cell membranes and involved in many physiological and biochemical functions, such as cell proliferation, differentiation, secretion, and the generation of volatile compounds [38,39]. In this study, the expression of 11 GPs was downregulated from Mth-6 to Mth-18 and Mth-30, which included PE(P-16:0e/0:0), PE(2:0/17:2), PE(3:0/16:1), PS(18:0/18:1), LysoPE 18:2, LysoPE 18:0, LysoPS 18:2, LysoPS 18:1, LysoPC 18:3, LysoPC 18:2, and LysoPC 18:1. Furthermore, the expression of eight GPs was highest in either Mth-18 or Mth-30, which included LysoPE 22:6, LysoPC 22:6, LysoPE 20:5, LysoPE 20:4, LysoPE 20:3, PE 21:5, LysoPE 22:5, and PE 19:1. The expression levels of these GPs were found to be higher in Mth-18 and Mth-30, demonstrating significantly elongated FA carbon chains and increased unsaturation compared to those highly expressed in Mth-6. This suggests that the carbon chains of the FAs contained in GPs were elongated in SAT from Mth-6 to Mth-18 and Mth-30; moreover, the unsaturation of the GPs increased with the elongation of the carbon chains. GP molecules containing very long and polyunsaturated FAs contribute to cellular proliferation, nutritional value, and a range of biological functions encompassing antioxidant activities, memory enhancement, and immunomodulation [7,40,41]. The results indicate that the GPs identified in Mth-18 and Mth-30 play a role in promoting SAT fat deposition in sheep, which is more beneficial for human health than in Mth-6. Concerning the glycerides, including monoglycerides, diacylglycerols, and triglycerides, non-absorbable triglycerides are converted into smaller absorbable molecules of monoglycerides and free FAs by the animal’s body. Monoelaidin, a monoacylglyceride, was found to be downregulated from Mth-6 to Mth-18, and its expression was lowest during Mth-18. It was suggested that monoelaidin was perhaps most absorbed during Mth-18. The analysis of fat thickness indicated that Mth-18 is the most metabolically active stage, characterized not only by faster lipid synthesis but also faster lipid absorption. Glycocholic acid is a sterol lipid, and is enriched in the bile acid biosynthesis pathway, acting as a primary bile acid [42]. Bile acids, primarily synthesized in the liver from cholesterol, can solubilize dietary fat and promote the absorption of fat and glucose homeostasis when they are secreted into the intestine [43]. Yang et al. [44] reported that bile acid promoted the hydrolysis of fats via lipases to release more FA, thus increasing energy expenditure in animals. Glycocholic acid was downregulated in Mth-18 and Mth-30, contributing to reduced fat expenditure in Mth-18 and Mth-30. The DE lipids were significantly enriched in alpha linolenic acid and linoleic acid metabolism and mitochondrial beta-oxidation of long-chain saturated fatty acids. During animal growth, there is a gradual decline in mitochondrial function, leading to a decrease in the β-oxidation of LC-FAs and an upregulation of LC-FAs expression [45,46,47]. Alpha-linolenic acid and linoleic acid metabolism is a process of FA desaturation and elongation; C20:5n-3 and C22:6n-3 are the main metabolic products of alpha-linolenic acid metabolism [48]. In this study, compared with Mth-6, the expression of C18:3n-3 was downregulated, while C20:5n-3 and C22:6n-3 were upregulated in Mth-18 and Mth-30, suggesting that the alpha-linolenic acid metabolism process was augmented in Mth-18 and Mth-30. 9,10,13-TriHOME is a metabolite generated through the lipoxygenase-catalyzed conversion of linoleic acid [49]. 9,10,13-TriHOME was downregulated in Mth-18 and Mth-30, suggesting a reduced oxidation capacity of linoleic acid during these growth stages. Fatty acid β-oxidation in mitochondria serves as a crucial pathway for regulating lipolysis and controlling lipid accumulation [50]. Acylcarnitines play a crucial role in facilitating the transportation of FAs across the mitochondrial membrane, serving as a pivotal intermediate in energy production through fatty acid β-oxidation [51]. The expression levels of stearoylcarnitine, palmitoylcarnitine, and acetyl-DL-carnitine were downregulated in Mth-18 and Mth-30 compared to Mth-6. This decrease in acylcarnitine expression may have resulted in a reduced capacity for fatty acid β-oxidation and energy expenditure, ultimately leading to an increase in the accumulation of LC-FAs and fat deposition observed in Mth-18 and Mth-30. Fatty acid amides play pivotal roles in diverse biological processes, encompassing the facilitation of lipid hydrolysis and the promotion of weight loss [52]. Fatty acid amides (including oleamide, palmitamide, and stearamide) were upregulated in Mth-18 and Mth-30, contributing a positive effect towards fat deposition. In conclusion, these DE lipids are responsible for differences in fat metabolism and have an impact on the fat deposition and quality of SAT.

In addition to lipids, non-lipid compounds also play a crucial role in the development of adipose tissue. Aspartic acid was found to be enriched in aspartate metabolism, the urea cycle, malate–aspartate shuttle, arachidonic acid metabolism, etc.; the metabolism pathways were related to amino acids, fatty acids, and energy metabolism. Thus, it may be a key metabolite for adipose tissue metabolism. Aspartic acid enters the TCA cycle after its conversion into oxaloacetic acid, thereby providing energy and carbon for de novo lipid synthesis [53]. Jersin et al.’s study [54] showed that more aspartic acid was required during adipocyte differentiation. Thus, the upregulation of aspartic acid in Mth-18 and Mth-30 may have contributed to enhanced umami flavor compound production and increased adipocyte proliferation and fat deposition. In addition, aspartic acid is a precursor of pyrimidine metabolism. Pyrimidine nucleosides play a crucial role in the biological processes of animals, such as promoting growth, improving immunity, and regulating the composition of amino acids and FAs [55]. In the pyrimidine metabolism pathway, cytidine-3’-monophosphate was upregulated in Mth-18 and Mth-30, suggesting that it could promote flavor formation. Glutathione (GSH) is a tripeptide composed of glutamate, glycine, and cysteine [56]. Asantewaa and co-workers [57] found that the loss of GSH decreased the expression of lipogenic enzymes, circulating triglyceride levels, and fat deposition. The expression levels of glutathione, L-glutathione (oxidized), and L-glutathione (reduced) were upregulated in Mth-18 and Mth-30, which may lead to fat deposition in these stages. In addition, sugar metabolism changed considerably in the SAT with the growth of the sheep. Studies with 3T3-L1 cells have revealed that morphological and cell composition changes were accompanied by significant shifts in glycolysis, oxidative pentose phosphate, etc. [58]. The expression of phosphoenolpyruvic acid, D-ribulose 5-phosphate, and acetic acid were upregulated in Mth-18 compared to Mth-6. Phosphoenolpyruvic acid was enriched in pyruvate metabolism, amino sugar metabolism, Warburg effect, and glycolysis/gluconeogenesis. D-ribulose 5-phosphate was enriched in both the Warburg effect and pentose phosphate pathway. Acetic acid was enriched in pyruvate metabolism and amino sugar metabolism. These results suggest that these metabolic pathways may be more active in Mth-18 than in Mth-6, contributing energy and precursor substances for lipid synthesis. Garcia-Galicia’s [59] study of lamb meat revealed that fat deposition (mainly UFAs) is promoted when the concentration of acetic acid increases as found in the current study. Phosphocholine plays a crucial role as an essential constituent of phospholipids in mammalian cell membranes, low-density lipoproteins, and lipid droplets [60,61]. The expression of phosphocholine is upregulated during sheep growth, thereby facilitating adipocyte proliferation and lipogenesis. Generally, the metabolite profiles in Mth-6 differed significantly compared to Mth-18 and Mth-30 and may be related to the growth performance of the sheep. The DE lipids and non-lipid compounds combine to regulate the metabolism of SAT. Acylcarnitines, fatty acid amides, aspartic acid, acetic acid, and phosphocholine may be key metabolites for regulating adipose tissue metabolism.

## 5. Conclusions

This study revealed the variation in fat thickness, adipocyte morphology, and metabolites of SAT in Sunit sheep at different growth stages. With the growth of the sheep, an increase in SAT deposition was observed due to adipocyte proliferation. Moreover, the carbon chains of FAs and lipids were elongated from Mth-6 to Mth-18 and Mth-30. The enrichment analysis revealed that the DEMs were predominantly enriched in key metabolic pathways, including glycerophospholipid metabolism, fatty acid synthesis metabolism, glycolysis/gluconeogenesis, aspartate metabolism, and others. Adipose tissue deposition may be correlated with the expression of metabolites, such as acylcarnitines, fatty acid amides, aspartic acid, acetic acid, and phosphocholine. The study revealed the variation in adipose tissue during the growth of naturally grazing sheep and explored the mechanism of fat metabolism. These findings will contribute to future studies on improving the edible quality of sheep meat and the development of the sheep industry.

## Figures and Tables

**Table 1:** Fatty acid compositions in subcutaneous adipose tissue of Sunit sheep in different growth stages (mg/100 g).

	Mth-6	Mth-18	Mth-30
C8:0	8.16 ± 0.36 ^a^	8.68 ± 0.86 ^a^	8.24 ± 0.28 ^a^
C10:0	139.62 ± 3.75 ^a^	86.83 ± 1.51 ^b^	91.44 ± 1.83 ^b^
C12:0	333.80 ± 59.03 ^a^	75.69 ± 8.48 ^b^	78.94 ± 7.70 ^b^
C13:0	24.80 ± 1.81 ^a^	13.74 ± 2.79 ^b^	14.08 ± 1.19 ^b^
C14:0	3406.00 ± 399.28 ^a^	1913.76 ± 206.22 ^b^	1941.31 ± 43.94 ^b^
C15:0	520.03 ± 25.69 ^a^	357.80 ± 54.98 ^b^	376.36 ± 15.74 ^b^
C16:0	13,629.55 ± 147.70 ^a^	13,150.13 ± 686.79 ^a^	1294.02 ± 175.82 ^a^
C17:0	911.50 ± 16.76 ^a^	1031.60 ± 10.67 ^b^	972.83 ± 13.35 ^c^
C18:0	9358.51 ± 186.43 ^a^	13,501.52 ± 132.74 ^b^	13,230.81 ± 441.67 ^b^
C20:0	36.82 ± 1.90 ^ab^	34.29 ± 4.94 ^a^	43.14 ± 1.77 ^b^
C21:0	57.00 ± 6.2 ^ab^	44.82 ± 1.42 ^a^	63.22 ± 8.83 ^b^
C22:0	15.50 ± 0.75 ^a^	7.36 ± 4.99 ^b^	24.66 ± 4.40 ^c^
C23:0	5.98 ± 0.77 ^a^	5.85 ± 0.41 ^a^	5.58 ± 0.33 ^a^
C24:0	47.40 ± 2.74 ^a^	51.91 ± 5.18 ^a^	48.21 ± 4.49 ^a^
C14:1	130.72 ± 9.47 ^a^	30.26 ± 2.64 ^b^	45.35 ± 0.40 ^c^
C15:1	25.03 ± 6.02 ^a^	58.22 ± 14.75 ^b^	49.93 ± 2.00 ^b^
C16:1	1454.34 ± 76.38 ^a^	1090.31 ± 127.17 ^b^	1226.24 ± 8.20 ^b^
C17:1	18.18 ± 1.19 ^a^	23.04 ± 0.7.84 ^a^	17.01 ± 2.04 ^a^
9t-C18:1	20.81 ± 3.65 ^a^	24.79 ± 4.89 ^a^	27.27 ± 2.92 ^a^
9c-C18:1	20,112.40 ± 944.72 ^a^	19,495.23 ± 405.04 ^a^	20,003.87 ± 555.85 ^a^
C20:1	443.73 ± 10.67 ^a^	456.78 ± 61.51 ^a^	448.31 ± 1.22 ^a^
C22:1	9.34 ± 0.97 ^a^	10.55 ± 1.76 ^a^	8.57 ± 0.05 ^a^
C24:1	14.06 ± 1.30 ^a^	12.44 ± 2.14 ^a^	12.51 ± 1.00 ^a^
9t,12 t-C18:2	190.81 ± 3.82 ^a^	182.79 ± 5.74 ^a^	187.92 ± 1.75 ^a^
9c,12 c-C18:2	726.44 ± 18.40 ^a^	711.98 ± 53.82 ^a^	673.75 ± 19.29 ^a^
C18:3 n-6	13.80 ± 0.37 ^a^	15.22 ± 2.33 ^ab^	17.18 ± 0.61 ^b^
C18:3 n-3	828.49 ± 3.23 ^a^	472.33 ± 38.33 ^b^	465.65 ± 22.34 ^b^
C20:2	7.20 ± 0.40 ^a^	6.22 ± 0.15 ^a^	7.51 ± 1.95 ^a^
C20:3 n-6	5.05 ± 1.36 ^a^	7.08 ± 2.98 ^a^	4.16 ± 0.46 ^a^
C20:3 n-3	39.22 ± 1.06 ^a^	42.04 ± 0.47 ^a^	42.53 ± 7.56 ^a^
C20:4 n-6	7.21 ± 0.56 ^a^	7.49 ± 0.56 ^a^	7.86 ± 0.89 ^a^
C22:2 n-6	5.26 ± 0.08 ^a^	5.48 ± 0.81 ^a^	5.35 ± 0.12 ^a^
C20:5 n-3	6.12 ± 1.00 ^a^	9.37 ± 1.01 ^b^	13.56 ± 1.63 ^c^
C22:6 n-3	12.76 ± 0.56 ^a^	19.18 ± 3.30 ^b^	15.08 ± 2.33 ^ab^
SFA	28,492.68 ± 268.60 ^a^	30,283.97 ± 801.37 ^b^	29,861.85 ± 590.09 ^b^
MUFA	22,228.62 ± 965.56 ^a^	21,201.61 ± 478.54 ^a^	21,839.05 ± 563.52 ^a^
PUFA	1842.35 ± 21.89 ^a^	1479.19 ± 69.20 ^b^	1440.55 ± 53.10 ^b^
PUFA/SFA	0.06 ± 0.00 ^a^	0.05 ± 0.00 ^b^	0.05 ± 0.00 ^b^
n-6	948.56 ± 19.09 ^a^	930.04 ± 60.65 ^a^	896.22 ± 21.27 ^a^
n-3	886.58 ± 3.93 ^a^	542.92 ± 40.81 ^b^	536.82 ± 33.43 ^b^
n-6/n-3	1.07 ± 0.02 ^a^	1.71 ± 0.05 ^b^	1.67 ± 0.07 ^b^

Data are presented as mean ± standard deviation. Different small letters in the same row indicate significant differences (*p* < 0.05).

## Data Availability

The data presented in this study are available on reasonable request from the corresponding author.

## Supplementary Materials

The following supporting information can be downloaded at: https://www.mdpi.com/article/10.3390/foods13040544/s1, Table S1: Principal component analysis of the metabolites.
